# Liver metastasis mimicking an abscess

**DOI:** 10.1259/bjrcr.20200201

**Published:** 2021-04-12

**Authors:** Kaoutar Imrani, Tlaite Oubaddi, Hounayda Jerguigue, Rachida Latib, Youssef Omor

**Affiliations:** 1 Department of Radiology, National Institute of Oncology, Mohammed V University, Rabat, Morocco

## Abstract

Hepatic metastases from carcinoid tumours are typically solid, hypervascular lesions on imaging. The cystic form, mimicking an abscess, is extremely rare. We report a case of a 48-year-old female presenting with a large hepatic mass that was diagnosed as a hepatic abscess, but the ultrasound-guided biopsy showed well-differentiated grade 1 neuroendocrine tumour. CT scan of chest, abdomen and pelvis was performed, looking for the primary tumour, it revealed an endobronchial mass of the right inferior lobe. Lung biopsy by rigid bronchoscopy was taken confirming the diagnosis of a typical carcinoid tumour.

## Case presentation

**Figure 1.**
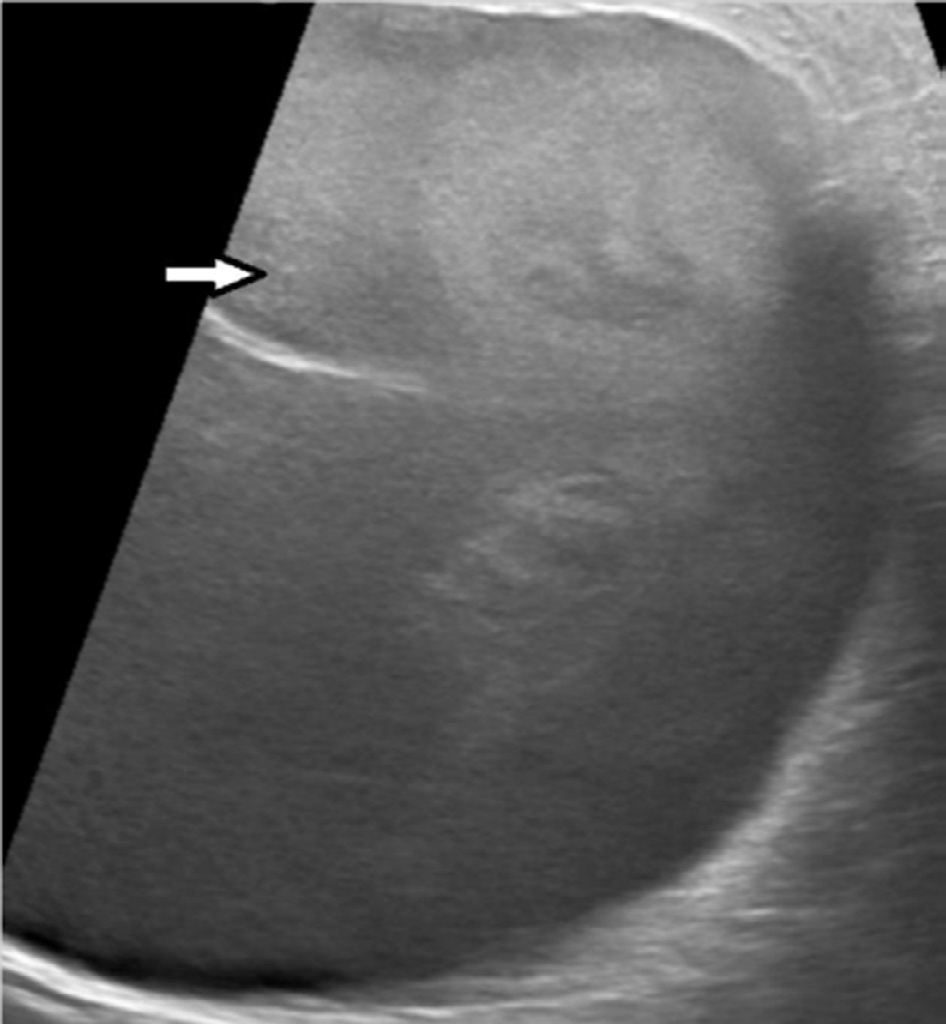
Abdominal ultrasonography showing a large well-defined, round cystic liver lesion with hypoechoic centre and an irregular wall

**Figure 2.**
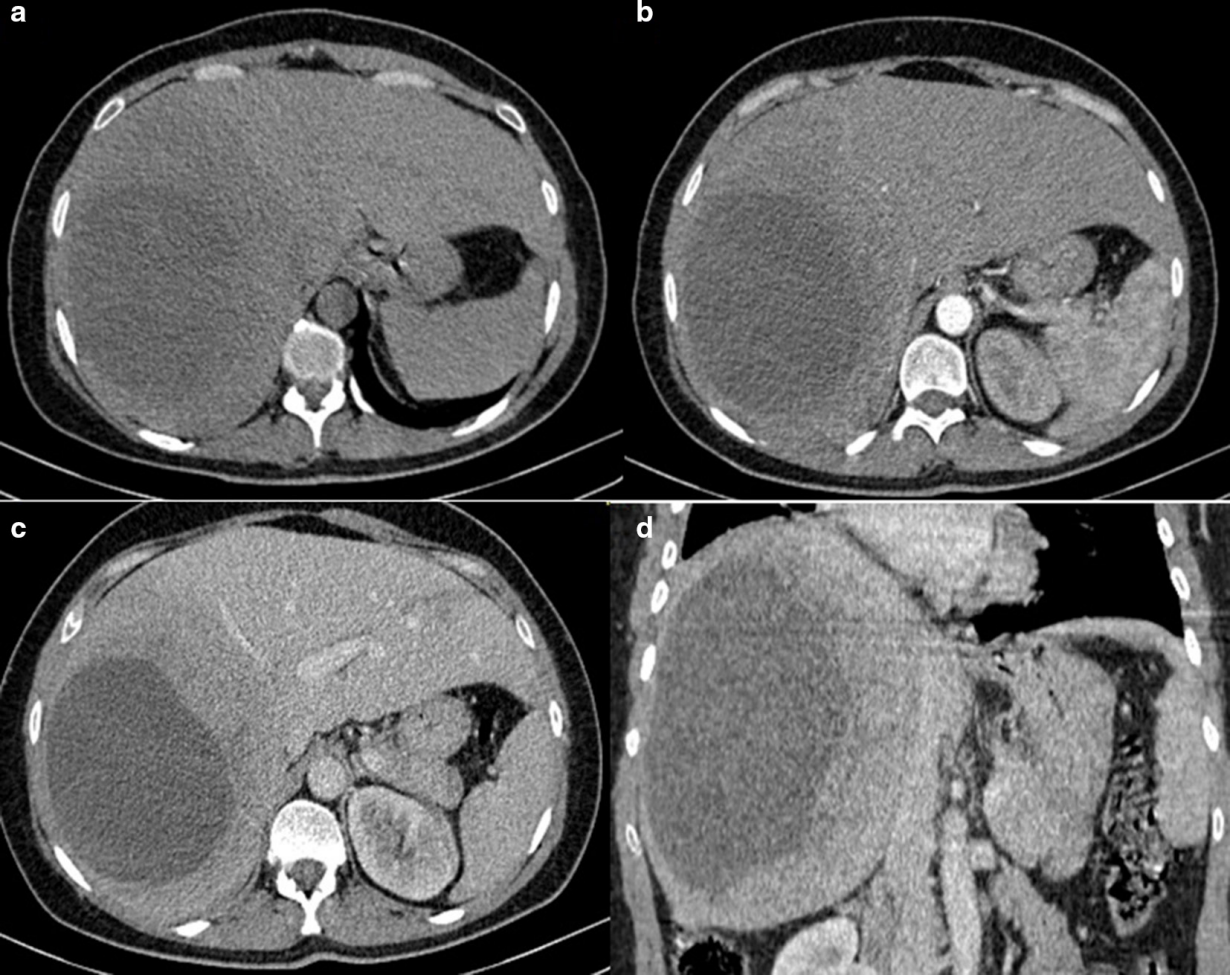
(**A**) Non-contrast CT scan showing a large hypodense lesion with thick wall in the right lobe of the liver (arrow). (**B**) Contrast-enhanced scan on arterial phase shows irregular peripheral enhancement (arrow).(**C-D**) Contrast-enhanced scan on portal phase shows no significant change (arrow).

**Figure 3.**
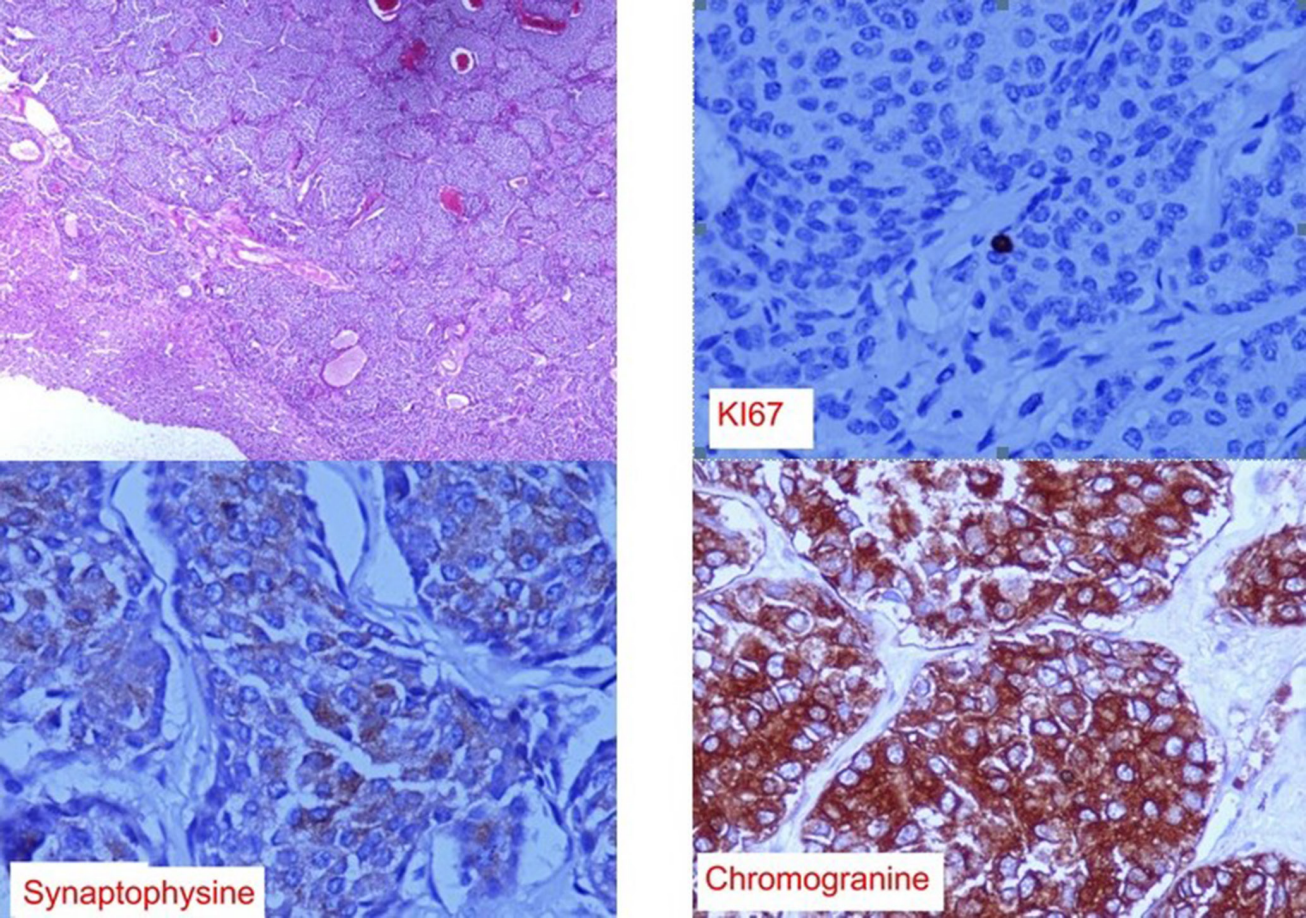
Histopathological examination of the hepatic biopsy: (upper left) hepatic infiltration by tumour proliferation with lobular and trabecular architecture. (Original magnification x 4) – Marking of very rare nuclei by the Ki67 proliferation index (original magnification x 40). Immunohistological staining (original magnification, 40 ×) is positive for synaptophysin and chromogranin and negative for CDX2 antibodies.

**Figure 4.**
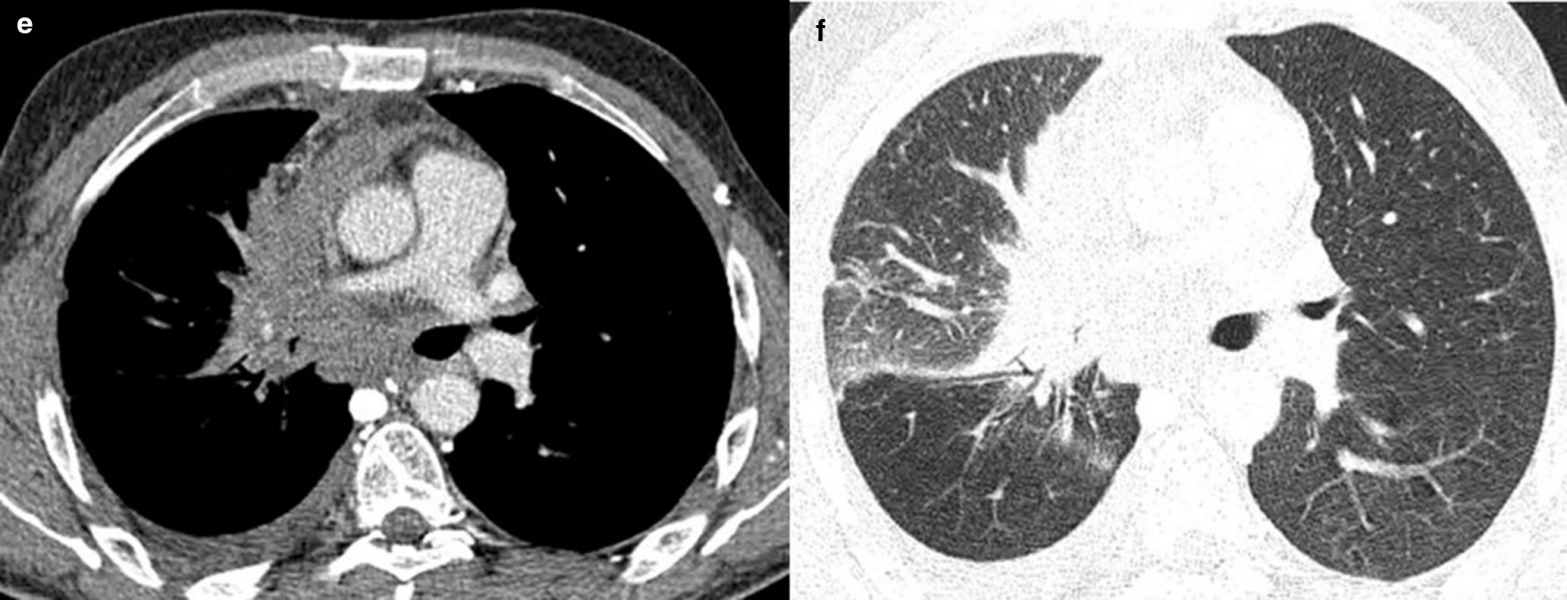
(**E-F**) Contrast-enhanced chest CT scans in mediastinal (**E**) and lung window (**F**) showed mildly enhancing irregular right hilar mass (arrow).

A 48-year-old female patient, with no medical history, presented to the emergency department for upper abdominal pain evolving for one month associated with vomiting, fever and chills, without other digestive disorder. The patient also reports a chronic paroxysmal cough with moderate dyspnoea. On physical examination, the patient had hepatomegaly with an enlarged mass. Laboratory data showed high range of white blood cell (13000 / µL) and a C-reactive protein value of 70 mg/L. Abdominal ultrasonography showed a large, well-defined, round cystic lesion occupying the entire right lobe of the liver, with hypoechoic center and an irregular wall (**Figure 1**). Contrast-enhanced abdominal CT scans at the arterial and portal phase revealed a well-defined, round-cystic lesion (Density of 18 HU) occupying the entire right lobe of the liver, measuring 18 × 13 × 19 cm, having a thick enhancing wall suggestive of a liver abscess (Figure 2). Ultrasound-guided biopsy of the larger liver mass were done using a Menghini needle 18 Gauge. Aspirate and biopsy cultures were negative for any growth. Aspiration cytology showed a chocolate coloured fluid, with epithelial cells, without atypia. Hepatic biopsy revealed a well-differentiated grade 1 neuroendocrine tumour, immunohistochemical staining was positive for synaptophysin and chromogranin and a Ki-67 labeling index <1% (Figure 3). Chest CT scans were performed revealing mildly enhancing irregular right hilar mass (Figure 4). Bronchoscopic biopsy of the hilar mass was performed using a transbronchial needle aspiration 19 Gauges, which confirmed the diagnosis of typical carcinoid tumour. The patient started a poly-chemotherapy based on VP 16/platinum, anthracycline cyclophosphamide but was lost to follow-up.

## Discussion

Hepatic metastasis may mimic a liver abscess, either because of the complex symptoms or due to indeterminate imaging features. This has been reported with colorectal cancers and neuroendocrine tumours (NET), especially carcinoid tumours, which originate commonly in the gastrointestinal tract or in other locations such as the lung, kidneys or the ovaries.^1^

Bronchial carcinoids, as in our case, account for 2% of primary lung tumours and approximately 25% of all carcinoid tumours.^2^ These tumours can be classified as typical or atypical based on their histological features.^3^ Atypical carcinoid has a more aggressive biological behaviour than typical one with a high risk of recurrence and distant metastases.^4^ Metastases occur in 15% of typical carcinoid tumours and 30–50% of atypical carcinoid tumors of the lung, and usually involve the liver, bone, adrenal glands and brain.

In this article, we report a case of liver metastases from typical carcinoid tumours of the lung, mimicking a liver abscess.

Liver metastases do not have pathognomonic symptoms, they are often silent. The common presentation of liver abscess includes fever, chills and right upper quadrant pain. These symptoms may be either present in liver metastases, because of secondary infection or superinfection of spontaneous necrosis.^5^

In the absence of pathognomonic symptoms, imaging, especially CT and MRI help the diagnosis. The typical CT imaging features of hepatic abscess are the presence of air bubbles or air-fluid level, double-target sign enhancement. Some characteristic CT findings may help differentiate between hepatic abscess and mass. Abscess shows inner air density, multilayered enhancement, double target or cluster sign, sharp margin and air biliary gram. It can be associated with pleural effusion, atelectasis of the lower lungs. However, a liver mass such as cholangiocarcinoma and hepatic metastasis would have a solid component with peripheral rim-like enhancement. It usually manifests with capsular retraction, focal bile duct dilatation and associate lymph node enlargement.^5,6^

Liver metastases of NETs, as in our case typically appear as solid, hypervascular masses with marked contrast-enhancement in the arterial and portal phases.^7^ The arterial hyperenhancement is due to a rich hepatic arterial supply. They may develop central necrosis or cysts, in this case, imaging features are atypical and can mimic liver abscesses in the pyogenic phase.^8,9^

The appearance of liver metastases on MRI can also mimic hepatic abscesses, they both appear hypointense on T1W images and hyperintense on T1W images with wall enhancement. Therefore, the role of apparent diffusion coefficient (ADC) calculated from diffusion-weighted imaging (DWI) allows the characterization of benign and malignant lesions.^9,10^

The necrotic portion of a tumour has a low viscosity and, therefore, a high ADCs. In contrast, the necrotic portion of an abscess cavity is filled with pus, which contains inflammatory cells, bacteria, necrotic tissue and proteinaceous exuded plasma with a very high viscosity and cellularity, thereby resulting in markedly decreased ADCs.^10^

Because of non-specific radiological features, percutaneous biopsy with histopathology studies may be required for a definitive diagnosis.^8,9^Fine-needle aspiration (FNA) is considered the gold standard with a yield that is highly sensitive. However, the process of fine-needle aspiration and its interpretation is fraught with risks. One study reported superiority of needle biopsy over FNA cytology allowing the demonstration of better architectural, cellular as well as immunohistochemical evaluation of liver lesions in biopsy specimens.^10^

## Conclusion

In the absence of typical clinical symptoms and because of non-specific radiological features, the diagnosis of liver metastasis and its differentiation from hepatic abscesses may still be challenging, especially in patients with unknown malignancy. Percutaneous biopsy is essential for a definitive diagnosis.

## Learning points

- The appearance of hepatic metastasis in CT scans can mimic hepatic abscess.
- It is important to make and consider the differential diagnosis to guide the therapeutic strategy.
- MRI and especially diffusion-weighted sequence have an important role to differentiate hepatic metastasis from abscesses.
- In the absence of a definitive diagnosis, percutaneous biopsy with histopathological studies may be required.

## References

[1] Banerjee A, Shah S. Liver tumors presenting as hepatic abscesses: a case series. HPB 2016; 18: e314: e314. doi: 10.1016/j.hpb.2016.02.810

[2] Modlin IM, Lye KD, Kidd M. A 5-decade analysis of 13,715 carcinoid tumors. Cancer 2003; 97: 934–59. doi: 10.1002/cncr.11105 12569593

[3] Salamone L, McCarthy S, Salem RR. Atypical cystic carcinoid tumors of the liver. J Clin Gastroenterol (2010; 44: e256–9 44.10. doi: 10.1097/MCG.0b013e3181da7714 20463590

[4] Filosso PL, Ruffini E, Oliaro A, Papalia E, Donati G, Rena O. Long-Term survival of atypical bronchial carcinoids with liver metastases, treated with octreotide. Eur J Cardiothorac Surg (2002; 21: 913–7 21.5. doi: 10.1016/S1010-7940(02)00055-6 12062286

[5] Mortelé KJ, Ros PR. Cystic focal liver lesions in the adult: differential CT and MR imaging features. Radiographics 2001; 21: 895–910. doi: 10.1148/radiographics.21.4.g01jl16895 11452064

[6] Seong NJ, Lee JM, Kim SH, Han JK, Kim YJ, Kim JH, et al. Differentiation between mass-forming type peripheral cholangiocarcinoma and hepatic abscesses: application of artificial neural networks to CT images. J Korean Radiol Soc 2005; 53: 343–52. doi: 10.3348/jkrs.2005.53.5.343

[7] Morikawa K, Igarashi T, Misumi S, Fukuda T, Ojiri H, Matsudaira H, et al. A case of pseudocystic liver metastases from an atypical lung carcinoid tumor. Radiol Case Rep 2019; 14 pp.: 595–601. doi: 10.1016/j.radcr.2019.02.022 30891110 PMC6406078

[8] Wang N, Ju Y, Wu J, Liu A, Chen A, Liu J, et al. Differentiation of liver abscess from liver metastasis using dual-energy spectral CT quantitative parameters. Eur J Radiol 2019; 113 pp.: 204–8. doi: 10.1016/j.ejrad.2019.02.024 30927948

[9] Akhondi H, Sabih DE. Liver abscess. In StatPearls [Internet. StatPearls Publishing 2020;. 30855818

[10] Taouli B, Koh D-M. Diffusion-Weighted MR imaging of the liver. Radiology 2010; 254 pp.: 47–66. doi: 10.1148/radiol.09090021 20032142

